# Real-time traffic sign recognition based on a general purpose GPU and deep-learning

**DOI:** 10.1371/journal.pone.0173317

**Published:** 2017-03-06

**Authors:** Kwangyong Lim, Yongwon Hong, Yeongwoo Choi, Hyeran Byun

**Affiliations:** 1 Department of Computer Science, Yonsei University, 50 Yonsei-ro Seodaemun-gu, Seoul, Republic of Korea; 2 Department of Computer Science, Sookmyung Women’s University, 47 Cheongpa-ro Yongsan-gu, Seoul, Republic of Korea; Beihang University, CHINA

## Abstract

We present a General Purpose Graphics Processing Unit (GPGPU) based real-time traffic sign detection and recognition method that is robust against illumination changes. There have been many approaches to traffic sign recognition in various research fields; however, previous approaches faced several limitations when under low illumination or wide variance of light conditions. To overcome these drawbacks and improve processing speeds, we propose a method that 1) is robust against illumination changes, 2) uses GPGPU-based real-time traffic sign detection, and 3) performs region detecting and recognition using a hierarchical model. This method produces stable results in low illumination environments. Both detection and hierarchical recognition are performed in real-time, and the proposed method achieves 0.97 F1-score on our collective dataset, which uses the Vienna convention traffic rules (Germany and South Korea).

## Introduction

As autonomous driving technology becomes important for upcoming car industries, fundamental driving assistant skills are being included in commercial cars and the size of the market is increasing. To create true self-driving technology and evolve from Advanced Driving Assistant System (ADAS) to Auto traveling, understanding the environment is important.

Currently alongside ADAS technology, another core technology for autonomous driving is Traffic Sign Recognition (TSR), a system which detects and recognizes traffic signs captured by a front mounted camera. With TSR, self-driving cars can thoroughly understand road regulations for safety. The European New Car Assessment Programme (EURO NCAP) regard TSR as top issue in safety skills for car safety [1]. In Germany, they host a TSR benchmarking competition [2, 3] to support academic and industrial development.

While ADAS requires an extra sensor such as radar or ultra-sonic equipment, TSR only requires a camera and machine learning technology. In order to execute TSR in real-time and with high performance, cutting edge hardware mounted in the car system is usually required. However, the recent announcement of NVIDIA’s General Purpose Graphics Processing Unit (GPGPU), DRIVE PX [4], a GPU for car systems, enables real-time heavy machine learning programs to run in the vehicle. Therefore, we propose a real-time accurate TSR system that leverages a GPGPU.

Object detection in driving scenes faces unique technical challenges. One major problem is wide variation in illumination due to weather and time of day. Another problem is low image quality at high speeds. To reduce the impact of these problems, various image pre-processing methods have been introduced; however, they come up short as they are not real-time procedures.

TSR technologies also use various methods to process the image. TSR is commonly a two step process: it first extracts the candidate region of the traffic sign and then classifies the detected region. For traffic sign detection, most methods are based either on the sign’s color or shape information [5–11] or on learning methods [12–18].

Color-based traffic sign detection uses the characteristics of traffic signs, which have limited and solid colors. These methods first segment specific colors to create a color map [5, 6, 7, 8] or a saliency map [9]. Traditional methods first find red or yellow areas and then finalize the candidate regions. The advantage of these methods is computational efficiency. However, these methods are largely impacted by illumination, meaning that performance accuracy decreases at night or in bad weather conditions (cloudy, rainy, etc.). To handle this issue, Y. Zheng suggest to reduce the illumination by improving the input images [19]. Shape-based traffic sign detection uses the limited shapes of traffic signs [8–10], i.e., circles, triangles, and octagons. These shape-based methods only work when the traffic sign shape is clearly presented in the image. Therefore, performance decreases when the shape becomes less clear, such as at night, under distortion, and under blurring.

Major learning-based traffic sign detection methods use Haar-like features and AdaBoost-based detection [13, 14] and deep learning based approaches [15]. The method of P. Viola and M. Jones first extracts the Haar-like features from an input image and constructs weak cascaded classifiers, which are assembled into one strong classifier and used to identify the traffic sign candidate region [13]. The advantage of P. Viola’s method is that, because it uses a cascaded classifier, the detection runs fast and the detection accuracy is fair. However, this method has several drawbacks. First, as the number of sign categories grow, both training and testing speed decrease when adding classifiers to the existing model. Second, if the input image includes significant noise or is recorded at night, the performance of the classifier becomes unstable. Finally, based on the characteristics of the cascade model, their method is not suitable for parallel processing on a GPGPU.

D. Ciresan et al. presented a traffic sign recognition method using a Convolutional Neural Network (CNN) [15]. Because it is designed for traffic sign recognition, the input image is not an actual driving scenes but a candidate region for the traffic sign. However, this method met the German Traffic Sign Detection Benchmark [2], and included a modified method to recognize diverse traffic signs following the German Traffic Sign Recognition Benchmark [3]. However, this system is based only on a candidate region for a traffic sign, whereas using a full driving scene as the input has not been tested. For deep-learning-based traffic sign detection, A. Møgelmose et al. presented Aggregated Channel Feature (ACF) [18], which is an improved version of Integral Channel Feature (ICF) [17] to train the deep learning model to detect and recognize U.S traffic signs. For this method, instead of an image pyramid they used a data-driven fixed scale feature pyramid and diverse channel features in their deep learning model. However, because of the limitations of the channel features (i.e., infeasible under low illumination) their recognition method performs well only during the day.

In this paper, we present the Byte-MCT and a landmark-based AdaBoost classifier for traffic sign detection. The presented method takes a driving scene as its input image and detects a candidate region using one classifier. After verifying the candidate region using a Support Vector Machine (SVM), the method recognizes the traffic sign using a CNN. This method uses Byte-MCT, a modified version of Modified Census Transform (MCT), which is robust against illumination variation. This method also utilizes a landmark feature, which allows the characteristics of the traffic sign to overcome partial occlusion of the input image. Additionally, during traffic sign detection, the method uses parallel processing (which can be implemented on a GPGPU) and reduces the detection time as compared to the sliding-window searching method. During the recognition stage, we use a cascade classifier that assembles the SVM to verify the candidate region and a CNN for diverse traffic sign recognition.

In the following section, we provide an overview of the presented method. Section 3 describes traffic sign detection that is robust to illumination. Section 4 describes the verification stage using SVM and recognition of traffic signs using CNN. Section 5 presents our experiment results for the testing set. Conclusions are given in section 6.

## Overview of proposed methods

Recently in the field of traffic sign detection and recognition, diverse methods have been introduced. However, several problems still remain to be solved.

1. The models still lack invariance to illumination; conventional models are optimized primarily for daylight scenes
2. Technical difficulties remain in real-time processing
3. Detection of diverse traffic sign requires multiple detectors

**Fig 1:**
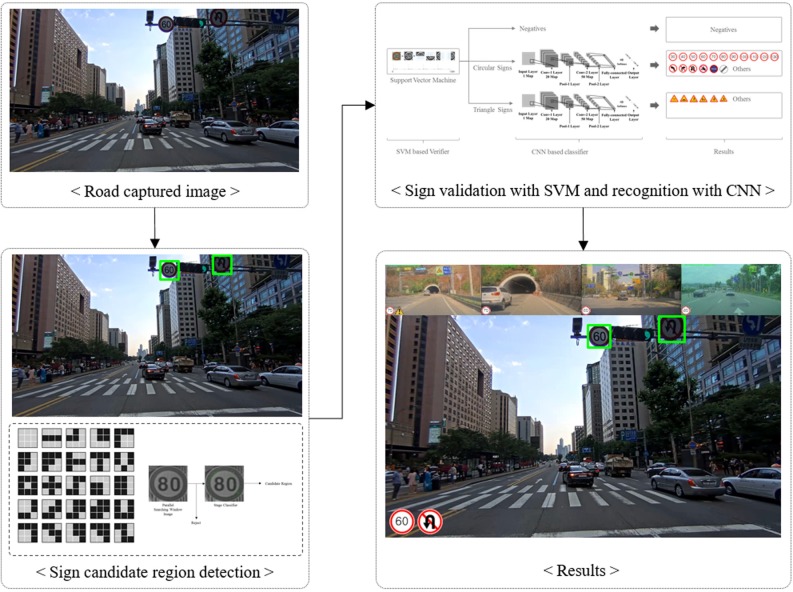
Block diagram of proposed method.

In this paper, we propose a GPGPU-based real-time traffic sign detection and recognition method to address each of these issues. The overall flow of the method is presented in Fig 1. First, we extract the Byte-MCT feature from the input driving scene to detect the candidate region. Unlike AdaBoost-based approaches [12–14] which use multiple sliding-windows for each shape such as circle or triangle, the proposed method uses landmark-based parallel-windows to detect multiple traffic sign candidate regions with fair performance, even in low illumination environments or night scenes. Second, for real-time processing on high resolution input, we implement a GPGPU-based parallel window searching method instead of raster image scanning. Parallel-based searching operates on a GPGPU in parallel for each divided grid of the input image.

After the candidate region is extracted from the input image, we split each candidate window into four partial window regions and generate feature vectors which are concatenation of Byte-MCT histograms. Consequently, the feature vectors are fed into SVM verification procedure. Therefore, feature vectors can distinguish traffic signs in similar shapes. The SVM classifies the detected windows into three classes, i.e., *circle*, *triangle*, and *other*. In the final recognition stage, we use CNN to classify the diverse traffic signs to further improve the result.

## Sign detection

In this chapter, we introduce a method for detecting traffic signs with illumination-invariant Byte-MCT feature in real-time. Illumination-invariance is an important element for traffic sign detection due to two senses: 1) detecting objects in images captured in a moving vehicle involve change of illumination which makes detection even more challenging, 2) weather and timeline shifts causes change of illumination.

### Byte-MCT features

In a digital image, each pixel can be described using Eq (1):

I(x)=gL(x)R(x)+b, (1)

where *x* is a pixel in the image and *I*(*x*) represents pixel *x* intensity, *L*(*x*) and *R*(*x*) represent the luminance of a light source and surface reflectance, respectively, *g* and *b* are camera parameters. The intensity of pixels are decided by combination of luminance and reflectance. If luminance becomes constant or limited in certain circumstances, then Eq (1) becomes Eq (2), indicating that the intensity is only impacted by the reflectance,

I′(x)=R(x)+b, (2)

where *b* is the constant bias. Thus, the equation is only affected by the reflectance of the surface, regardless of the strength of luminance. The effect of luminance from input pixels can be ignored if we normalize the input pixel’s intensity. However, measuring the exact luminance from each pixel is infeasible. The closest approximation method is Census Transform (CT). The CT uses either 3-by-3 or 5-by-5 fixed-size windows to construct index of edge structure based on relative intensity comparison between middle index pixels and neighbor pixels. The most conventional CT methods are Modified Census Transform (MCT) and Local Binary Pattern (LBP).

B. Froba and A. Ernst [12] presented MCT, which uses only the reflectance from the digital input to perform CT to handle images with diverse luminance variance. For given a window of pixels, the MCT converts each pixel into binary value by comparing it with the average intensity. If the pixel is brighter than the average, the method transforms the pixel into a 1; otherwise, it is 0. For example, for a 3-by-3 window, the MCT has a 9-bit binary pattern. However, unlike CT, which uses the overall average pixel intensity, MCT uses average pixel intensity for only local region. Therefore, MCT is more robust against low illumination inputs. Eq (3) shows an MCT operation on a pixel *x*,

$$\Gamma (x)={⊗}_{y\in N}I\;(I_{avg}(x)<I(y)),$$ (3)

where *N*(*x*) is the eight neighboring pixels of *x*, *I*_*avg*_(*x*) is average intensity of *N*(*x*), and I is an indicator function that yields 1 if the statement is true and 0 otherwise. ⊗_*y*∈*N*_ is a concatenation of neighboring pixels’ transformation result. As a result, MCT can describe up to 511 (= 2^9^−1) local edge structures in a 3-by-3 window. Since MCT compares each pixel to the average intensity of local region, global illumination has no impact and only reflectance is considered thus it becomes robust against luminance change.

**Fig 2:**
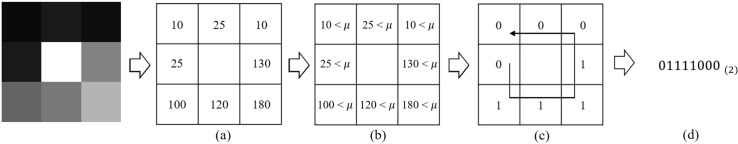
Example of the Byte-MCT code generation.

In this paper, we further modify MCT method to present Byte-MCT (8-bit MCT). Byte-MCT excludes the central pixel from concatenation, resulting in only eight neighbor pixels. Fig 2 shows an example of Byte-MCT procedure. This transform gives two advantages over 9-bit MCT [12]. First, computational efficiency; while preserving the most of feature descriptors from 9-bit MCT, such as straight line, diagonal line, corner, etc., it reduces the feature vector size into 255 dimensions, which allows faster learning. Second, robustness to noise; the proposed Byte-MCT only considers the difference between eight neighbors in 3-by-3 region and their average. It leads to the robustness to the noise in the center pixel, since the center pixel does not affect the feature descriptor unlike 9-bit MCT. The effectiveness of Byte-MCT will be discussed in the experiment sections.

### Landmark-based AdaBoost training

In this section, we describe a method of identifying traffic sign landmark points using Byte-MCT. Y. Freund and R. Schapire [20] introduce a cascade algorithm called AdaBoost. The AdaBoost algorithm constructs multiple weak classifiers and assembles them into a single strong classifier for the overall task. AdaBoost’s main strength lies in its high prediction power and capability to cascade an architecture for improving the computational efficiency; thus, it is commonly used in the object detection techniques. B. Froba et al. trained AdaBoost to detect the face region using MCT. The method first generates the same number of weak classifiers as the number of pixels and then assembles a strong classifier, which has four stages. The method assigns high weights to good weak classifiers and low weights if the classifier causes a large error. Because the method generates the same number of weak classifiers as input pixels, an image that is similar to the training data will be well classified for similar regions. However, if the input data have wide variations or abrupt changes in background, then the training time increases and classifier accuracy decreases. To handle this limitation, we modify AdaBoost method, to present landmark-based AdaBoost.

Table 1 shows the algorithm of landmark-based AdaBoost classifier. *D*_1_(*i*) is the initial weight of the first pixel classifier; *i* indicates the sign or non-sign and *D* is the boosting weight. After assigning the initial boost weight to construct the strong classifier, we train the assigned number of pixel classifiers. During pixel classifier training, each trained class generates a Byte-MCT-based look-up table and calculates the pixel classifier weight α based on error ε. In each learning iteration, the weak (pixel) classifiers are trained and assembled into a strong classifier. The learning iteration ends when the overall sum of weak classifier error is less than *θ*_*t*_ and *θ*_*α*_ is bigger than α, because α represents the importance of the weak classifier.

**Table 1:** AdaBoost learning algorithm based on landmarks.

*i) input sign and background training images*	
*ii) Calculate initial boosting weight distribution*	
	*D*_1_(*i*) = 1/*m*
	*Where*, *i indicates sign (= 1) or background (= 0) and m is number of total images*
*iii) for t = 1*, *… T*	
	*do loop* {
	*- Generate look-up table ω*_*t*_ *from minimum ϵ*_*t*_
	$\varepsilon_{t}=\sum_{x\in X}min(g_{t}^{0}(x),g_{t}^{1}(x))$
	$g_{t}^{0}(x,\gamma )=\sum_{i,x,\gamma }D_{t}(i)\;I(\Gamma_{i}(x)=\gamma )\;I(c_{i}=0)$
	$g_{t}^{1}(x,\gamma )=\sum_{i,x,\gamma }D_{t}(i)\;I(\Gamma_{i}(x)=\gamma )\;I(c_{i}=1)$
	*Where*, *function I() is the indicator function and*
	*Γ*_*i*_(*x*) *output Byte-MCT kernel index at x and c*_*i*_ *is image class*
	*- Calculate alpha*
	$\alpha_{t}=\frac{1}{2}ln(\frac{1-\varepsilon_{t}}{\varepsilon_{t}})$
	*- Update distribution*
	$D_{t+1}(i)=\frac{D_{t}(i)}{Z_{t}}\times \left\{ \begin{array}{l} e^{-\alpha_{t}},\;if\;\omega_{t}(\Gamma_{i}(x))=c_{i}\\ \;e^{\alpha_{t}}\;,\;if\;\omega_{t}(\Gamma_{i}(x))\neq c_{i} \end{array}\right.$
	$\omega_{t}(\gamma )=\left\{ \begin{array}{l} 0,\;if\;g_{t}^{0}(x_{t},\gamma )>g_{t}^{1}(x_{t},\gamma )\\ 1,\;else \end{array}\right.$
	$Z_{t}=\sum_{i}D_{t}(i)$
	*- Check the loop stop condition*
	*if ∑_p∈P_ ε_i_ < θ_t_ and α_min_ > θ_α_ then Exit*
	}

B. Froba et al.’s method trains the pixel classifiers in four stages. At each stage, the classifier is trained until the error is less than the threshold value. The drawback of this method is that it trains all of the pixel classifiers until it reaches the end condition, meaning that the learning time sharply increases as the input resolution increases. To overcome this disadvantage, the modified AdaBoost method trains weak classifiers only on the landmark pixels. The values of landmarks are decided by α-score from Table 1. The *T* landmark pixel classifiers are weak classifiers which considers the most distinguishable pixel and are combined into the strong classifier. Unlike conventional ensemble classifiers, which use multiple stages (for time efficiency) instead of full training, the proposed method has only one strong classifier with a small number of landmark points. Because of the algorithm’s structure, the proposed method calculates far fewer operations than the original AdaBoost. Another benefit of the landmark-based architecture is that it can run on a GPU with parallel-window searching. The GPU’s parallel system divides one task into multiple small independent tasks that can be processed by thousands of GPU core blocks. Therefore, for non-sequential tasks such as sliding-window, this method can take advantage of the parallel system. Details will be presented in next section.

**Fig 3:**
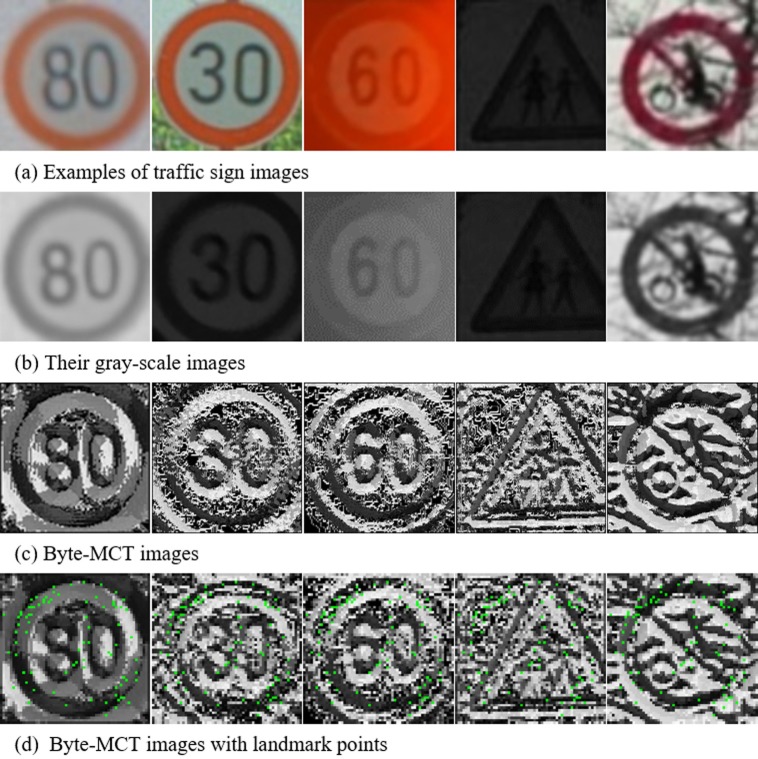
Example of landmarks in a traffic sign using the proposed AdaBoost learning algorithm.

Fig 3 shows the landmark-based AdaBoost learning method with Byte-MCT. In other words, we demonstrate a modified version of AdaBoost that focuses on identifying only important locations, i.e., landmarks.

### GPU-based real-time detection

The two major approaches for object detection are sliding-window and object-proposal. Object-proposal is based on edge shapes. Usually, object proposals extract up to 1,000 candidate regions per image, regardless of the shape of the object. Because traffic signs have distinct shapes, object-proposal is not a suitable method. For this reason, TSR methods commonly use holistic searching in sliding-window fashion.

Traditional sliding-window searching scans the entire image from a fixed window using raster scanning; thus, running time increases as the input resolution increases. To handle this challenge, the conventional methods construct a cascaded ensemble classifier using AdaBoost. The cascade method increases the running time efficiency by skipping non-object areas. However, this method requires sequential processing and cannot be run on a GPU. Therefore, object detection on a Full-HD image in real time is difficult.

**Fig 4:**
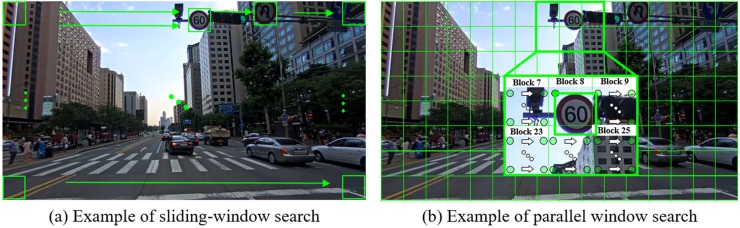
Examples of sliding-window search and parallel-window search.

Advances in modern hardware has transformed CPUs from a single-core sequential processors into multi-core multi-thread parallel processors. This parallel processing paradigm can also be applied to GPGPUs, which have more than thousands of cores in a single unit. In this paper, we propose a Byte-MCT and landmark-based AdaBoost classifier using a parallel window-searching method on a GPGPU as shown in Fig 4.

Unlike the sliding-window method, which scans an image in a sequential manner, parallel window-searching divides the input image into several blocks and simultaneously performs classification on one block using each GPU core. Although the GPU parallel system shares the concept of the CPU parallel system, GPU-based parallel window-searching has challenges with arbitrary positions in memory. We use a kernel function to address this challenge.

Table 2 describes the parallel window-searching method’s kernel function on a GPGPU. In the GPGPU, the kernel function does not have access to the host’s memory and is unable to return. Therefore, we generate an object map. The object map keeps the search window size and its exact location identified by the current kernel function. Unlike sequential searching, parallel searching implemented on a kernel function generates blocks based on the number of GPU cores and the H/W pipeline. Because each block simultaneously runs the kernel function, this method allows real-time processing. The introduced parallel window searching method takes 2.4ms on Full-HD (1920 × 1080) resolution images and using a 40-by-40 search window size, whereas the same input image takes 352.26ms using a CPU-based sliding window search. In other words, the GPGPU version is 140 times faster than the CPU version; details will be provided in section 5.

**Table 2:** Example of GPU kernel function.

__global__ void gpu_detectObjects(		
		int* gpu_mctFeat, int* gpu_objectMap, int mctWidth, int mctHeight,
		int roiWidth, int roiHeight, float mctThreshold,
		int *gpu_wq, float *gpu_th, int *gpu_xt,
		float *gpu_at, float *gpu_wt)
{		
	const int idx = blockIdx.x * blockDim.x + thread.x;	
	const int yPos = idx / mctWidth	
	const int xPos = idx % mctWidth;	
	const weakClassifierQty = gpu_wq[0];	
	float classifierThres = gpu_th[0] + gpu_th[1] * 0.48;	
	float sum = 0.0f;	
	for (int i = 0; i < weakClassifierQty; i++)	
	{	
		const int sub_x = xPos + gpu_xt[i] % roiWidth;
		const int sub_y = yPos + gpu_xt[i] / roiWidth;
		const int sub_pos = sub_y * mctWidth + sub_x;
		int sub_feat = gpu_mctFeat[sub_pos];
		sum + = gpu_at[i] * gpu_wt[(i*256) + sub_feat];
	}	
	if (sum < classifierThres)	
	{	
		gpu_objectMap[idx] = (int)roiWidth;
	} else {	
		gpu_objectMap[idx] = (int)0;
	}	
}		

## Sign recognition

In the previous section, the method for extracting the candidate region of the sign was described. In this section, we discuss SVM-based verification and CNN-based recognition. The recent emerging of deep learning network become one of main backbone of self-driving industries [21, 22]. However, the verification method reduces the number of candidate regions, since SVM has better optimization ability with the limited or fairly fewer number of inputs compare to CNN and tested in experiments section. The recognition method understands fine-grained information from verified input and decides final result.

### Sign verification using SVM

**Fig 5:**
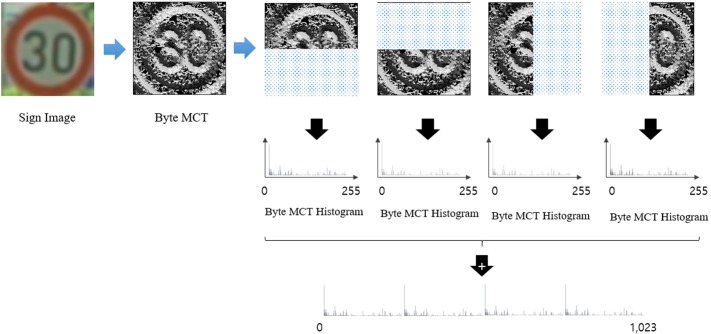
Example of verification of feature extraction.

To verify candidate regions extracted by AdaBoost, we adapt the Byte-MCT as feature vectors for SVM. The SVM determines the decision boundary that has a maximum margin from the support vector, which is an outlier of the feature vector. It verifies the candidate regions with better generalization ability than CNN. Fig 5 shows feature extraction for region verification. From the candidate regions, each pixel value represents the 3-by-3 Byte-MCT local edge structure that is used in verification. First, the structure is divided into four overlapping regions as in Fig 5. Each region’s Byte-MCT histogram stacks into 1,024-dimensional feature vectors. Eq (4) is the mathematical equation for the feature extractions;

$$H=\sum_{r=1}^{4}\frac{H_{r}}{max(H_{r})},$$ (4)

where *r* is the number of candidate regions and max(*H*_*r*_) is the maximum histogram value from the divided region. This equation normalizes each divided region using its own maximum value to generate the 256-dimensional histogram. By concatenating the 256-dimensional histograms, the method generates a 1024-dimensional feature vector. Because this feature extraction process uses Byte-MCT input, the extracted feature is also robust against illumination. Finally, the SVM calculates the extracted feature vector using the Radial Basis Function (RBF) kernel to classify the input classes: circle, triangle, and other.

The circle class indicates a prohibition sign, which has a red rim. The triangle class is a warning sign. The negative class is any place in which images from the input are not traffic signs. This class also includes background images that have similar shapes as traffic signs. In the real world, same traffic sign shapes have different meanings based on information inside the signs such as 40 mile speed limit and 60 mile speed limit have same shape but different restriction. Additionally, the background image can also contain shapes similar to traffic signs yet have no meaning in terms of traffic rules. This situation requires additional recognition steps for fine grained recognition to reduce the false positive inputs.

### Structured CNN-based sign recognition

**Fig 6:**
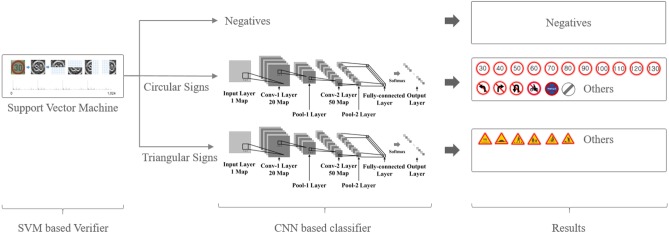
Example of entire sign recognition structure.

From an SVM-verified image, such as a circle or a triangle, we use CNN to recognize signs. In the German Traffic Sign Recognition Benchmark (GTSRB) [3], the CNN-based approach achieves state-of-art performance from diverse traffic signs via only one CNN. The GTSRB contest already has produced methods for generating cropped traffic sign regions. However, the goal of this paper is to recognize signs in real-world driving images, which include diverse shapes and types of traffic signs. From previously SVM-verified regions, the CNN classifies the information as shown in Fig 6. To reduce the number of false positive images (i.e., inputs that pass SVM verification but ought to fail), the presented CNN model includes one more class, i.e., *other*.

**Fig 7:**
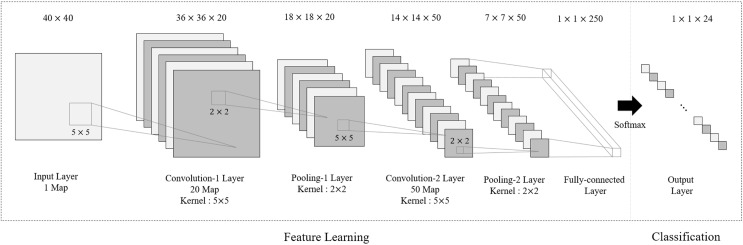
Example of proposed model architecture.

Fig 7 presents the CNN architecture model of each SVM class. Depending on the number of layers and the size of the kernel, a CNN can extract different features from the same input image. Normally, models with deep layers and large kernel sizes are suitable for mid-level feature extraction and are typically used for scene understanding. On the other hand, shallow-layer models are suitable for classification of a limited number of images. D. Ciresan et al. [15] who presented the highest accuracy model from the GTSRB, built a CNN model with three convolution layers and a pooling layer for traffic sign recognition that is robust against noise and occlusion.

The presented CNN model takes a 40-by-40 input image and generates 250-dimensional feature vectors from two pairs of convolution and pooling layer. This feature vector passes the fully connected neural network with the final softmax layer that classifies the 24 traffic sign types. Unlike D. Ciresan’s model [15], the proposed model has only two pair of layers, namely a convolutional layer and a pooling layer. A shallower architecture learns detailed features rather than high-level features, since the verification step reduces the number of input for each category.

## Experiments

### Datasets

To evaluate our proposed method, we use two different datasets: the LISA US traffic sign dataset [18] and a real-world driving dataset that records the driving scene directly from a car mounted camera. The LISA dataset contains driving scenes from the Southern California region, including US traffic signs, speed-limits, warnings, no-turns, stops, etc. The LISA dataset is based on different rules than the Vienna traffic rules. To test both scenarios, we collected driving scenes in two locations that apply the Vienna traffic rules: Korea and Germany.

**Fig 8:**
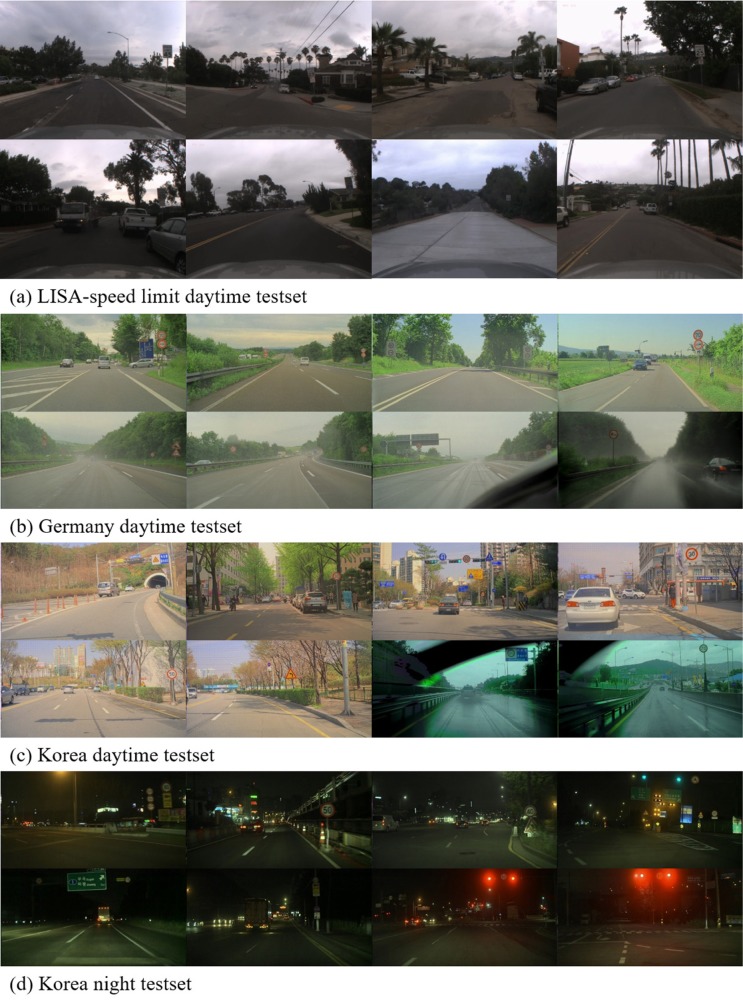
Examples from the LISA US speed-limit dataset and our real-world driving dataset.

Fig 8 shows examples of both the LISA speed-limit dataset and the recorded Vienna traffic rules dataset. The Vienna traffic rules dataset was recorded using a front-mounted High Dynamic Range (HDR) camera using an Electric Chromatic Mirror (ECM). The data is composed of three subsets: Korean daytime, Korean nighttime, and German daytime (KR-D, KR-N, DE-D, respectively).

Table 3 shows the specifications of the KR-D, KR-N, and DE-D datasets. All the frames in each dataset has HD (1280 × 672) resolution, and at least half of the frames contain traffic signs.

**Table 3:** Specification of test-sets.

Name	Type	Location	Frames	Signs	Resolution
LISA-SPEED	Daylight, Cloudy	USA	641	637	1280 × 960
DE-D	Daylight, Rain, Cloudy	Germany	4,765	2,001	1280 × 672
KR-D	Daylight, Rain, Cloudy	Korea	4,527	2,294	1280 × 672
KR-N	Night	Korea	3,086	1,343	1280 × 672

Fig 8(B) shows driving scenes under diverse weather conditions from both German metropolitan area and countryside in daytime. Fig 8(C) and Fig 8(D) show daytime and nighttime driving scenes in Korea under roughly the same weather and light conditions as in Germany. In order to test the model’s robustness against illumination changes, the KR-N dataset contains a large number of frames of heavy illumination changes in downtown scenes.

### Detection and recognition performance

To evaluate the presented model, we compare its performance to that of the Aggregated Channel Feature (ACF) method [18], which is ranked high on the LISA SPEED-LIMIT dataset. The ACF method’s performance for the recorded datasets under the Vienna traffic rules is shown in Table 4. This method only detects the candidate region; thus, the recognition part is missing. Table 5 shows both detection and recognition performance results for the presented model on the same datasets. For all datasets considered, the presented method is superior in both detection and recognition to the ACF method. In the tables, TP indicates true positive, which means correctly recognized object. FP indicates false positive, which means either detecting the wrong object or misrecognition with correct localization. FN indicates false negative, which means that the method was unable to detect the sign. As shown in Table 4, the ACF method achieves 81.9% and 84.3% accuracy for the daylight dataset and the LISA speed-limit dataset, respectively. Additionally, in the nighttime dataset, ACF achieves only 15.7% with 0.09 recall. Because the ACF method is based on traffic sign shapes, detection is hindered by nighttime or low illumination environments. Table 5 shows accuracy achieved by the proposed method, which are 97.8% and 89.5% in daylight sets and LISA speed-limit datasets, respectively. Additionally, even in the nighttime dataset, the presented method achieves 89.5% accuracy. Unlike ACF, which is based on traffic sign shapes, the presented method uses landmark detection for improved detection performance and Byte-MCT to allow stable performance, even in low-illumination situations. Table 6 shows accuracy achieved using single CNN. Considering Tables 5 and 6, proposed method achieve more stable performance as described in sign recognition section.

**Table 4:** Results of detection performance using ACF without classification.

Method	Dataset	TP	FP	FN	Precision	Recall	F1
ACF	LISA	N/A	N/A	N/A	N/A	N/A	0.8431
	DE-D	1,275	356	429	0.7817	0.7482	0.7646
	KR-D	1,739	323	233	0.8433	0.8818	0.8621
	KR-N	122	82	1,114	0.5773	0.0913	0.1577

**Table 5:** Results of detection and recognition performance using the proposed method.

Method	Dataset	TP	FP	FN	Precision	Recall	F1
Proposed	LISA	528	19	104	0.9652	0.8354	0.8956
	DE-D	1,916	47	73	0.9760	0.9633	0.9696
	KR-D	2,249	22	44	0.9903	0.9808	0.9855
	KR-N	1,314	28	19	0.9791	0.9857	0.9824

**Table 6:** Results of detection and recognition performance using the single CNN.

Method	Dataset	TP	FP	FN	Precision	Recall	F1
Byte-MCT Detector & CNN only	DE-D	1,632	361	53	0.8188	0.9685	0.8874
	KR-D	1,486	535	536	0.7352	0.7349	0.7351
	KR-N	1,190	937	195	0.5594	0.8592	0.6776

**Fig 9:**
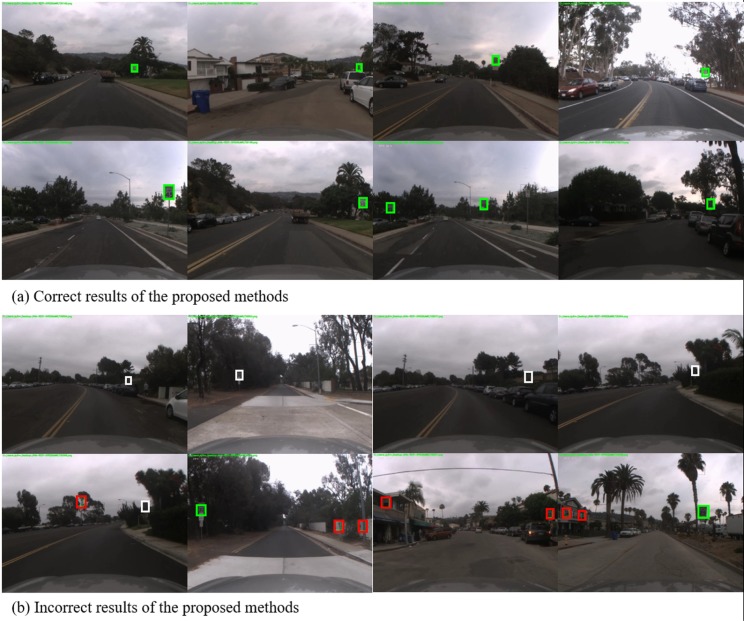
Resultant examples of proposed method for the LISA speed-limit test-set.

Fig 9 shows an example of proposed method’s results for the LISA dataset. Throughout all figures, the green box indicates the correctly predicted detection result, the white box indicates the missed signs, and the red box indicates the wrongly detected ones. Fig 9(A) shows the correctly predicted cases from small far-away traffic signs to large nearby traffic signs, regardless of the illumination difference. Fig 9(B) shows the false detection case in which the traffic sign is significantly rotated, the traffic sign size is too small, or the background is similar to the traffic sign.

**Fig 10:**
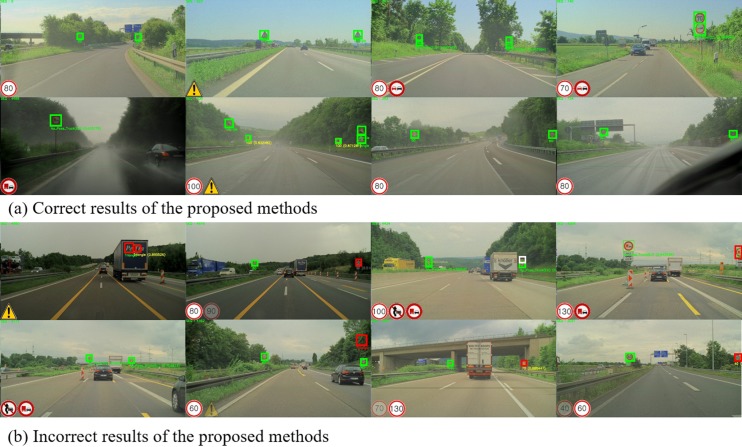
Resultant examples of proposed method for the Germany (DE-D) test-set.

Fig 10 shows the German daylight dataset (DE-D) results of the presented method. As Fig 10(A) shows, the presented method has stable performance for correct detection and recognition of the traffic sign under heavy rain and low illumination scenes. Fig 10(B) shows the failure cases in which the method could not detect or misinterpreted traffic signs. In DE-D dataset, false recognition rate is higher than that of KR-N dataset. We expect that this occurred due to camera blur caused by higher driving speed. The Autobahn highway has a much higher speed limit, faster driving can cause turbulence around the camera, which leads to further blur of the image.

**Fig 11:**
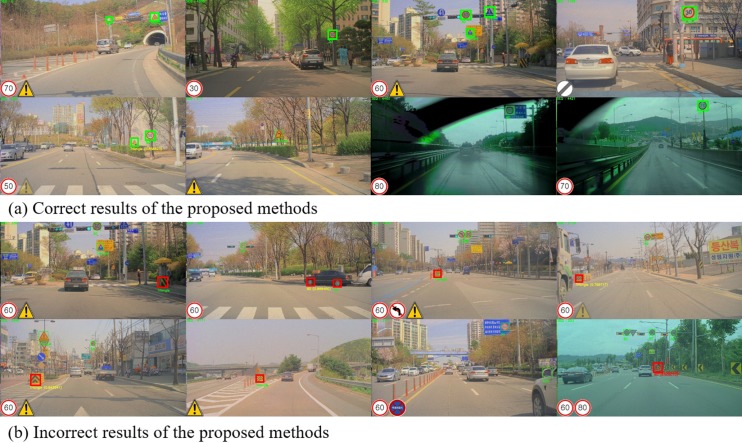
Resultant examples of proposed method for the Korea (KR-D) test-set.

Fig 11 shows results on the Korea daylight dataset (KR-D). Fig 11(A) shows the correctly predicted results in various situations. The proposed method detects and recognizes traffic signs that are both small or close to the car under heavy rain. The presented method has 0.9855 F1 score on the KR-D datasets, which means that most of the objects were detected and recognized. Fig 11(B) shows the failure cases. The KR-D dataset includes more metropolitan driving frames than DE-D. As the example shows, the major false positive is due to watery bumpers. Watery bumpers, which prevent or minimize vehicle accidents, are usually located on the median strip or close to bus stops. These bumpers have a similar color scheme and shape as triangle warning signs, which causes false detections.

**Fig 12:**
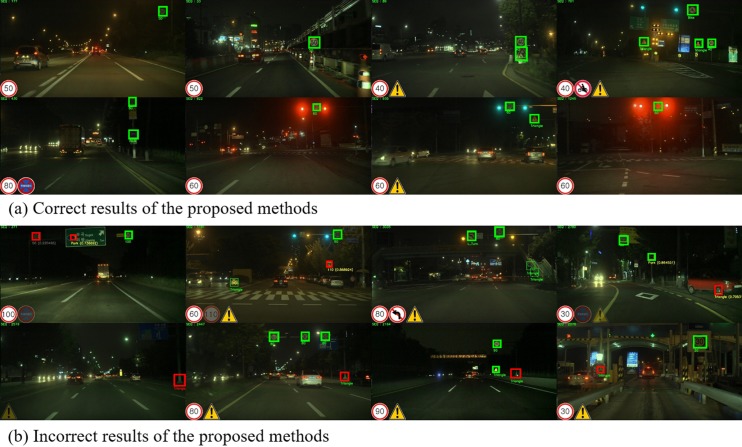
Incorrect results using the proposed method for the Korea (KR-D) test-set.

Fig 12 shows the Korea nighttime dataset (KR-N) results. Fig 12(A) shows the correctly predicted cases, which prove that the proposed method works under illumination changes due to low illumination, neon signs, and traffic lights. This scenario dataset usually results in failure cases for shape-based and color-based approaches. In Fig 12(A), in the last example, the traffic sign is located between traffic lights with red lights illuminated. This example is an extreme failure case for the shape-based approach, because the shape is difficult to identify; however, the proposed method is able to find the correct location. Fig 12(B) shows the failed cases. In nighttime scenes, based on camera settings, noise can be easily added to the image. Therefore, nighttime usually causes more false detections. Using SVM as a verification method, the presented method significantly reduces the number of false detections. However, there are still false detections after the verification steps. The false detections are mostly detected from triangular shapes, which can be easily fixed by post processing since they do not last more than one frame.

### Comparison of detection performance

To evaluate the effectiveness of proposed detection method, we conduct the experiments with two factors: MCT features, and AdaBoost. Since the focus of these experiments is comparing detection performance between features, cascade of architectures is excluded so that maximum performance is bounded by non-cascaded models. From Table 7, 9F is the reference method [12], 9-bit MCT with full- pixel based AdaBoost. 9L is 9-bit MCT with landmark based AdaBoost. 8F is Byte-MCT with full-pixel based AdaBoost. 8L is proposed method, Byte-MCT with landmark based AdaBoost. Among the experiments, the presented method shows the highest performance.

**Table 7:** Results of detection performance comparison.

Dataset	Detector	Recall
DE-D	9F (9bit MCT + Full-size classifier)	0.5476
	9L (9bit MCT + Landmark classifier)	0.8473
	8F (8bit MCT + Full-size classifier)	0.5678
	8L (8bit MCT + Landmark classifier)	**0.9967**
KR-D	9F (9bit MCT + Full-size classifier)	0.8236
	9L (9bit MCT + Landmark classifier)	0.9795
	8F (8bit MCT + Full-size classifier)	0.8304
	8L (8bit MCT + Landmark classifier)	**0.9851**
KR-N	9F (9bit MCT + Full-size classifier)	0.5102
	9L (9bit MCT + Landmark classifier)	0.7074
	8F (8bit MCT + Full-size classifier)	0.5355
	8L (8bit MCT + Landmark classifier)	**0.9885**

9-bit MCT and full-pixel AdaBoost, shows superior precision compared to Byte-MCT with full pixel based AdaBoost regardless of datasets. However, the performance of features are decided by recall, since detection performance is thoroughly influenced by choosing true positive. Unlike precision, recalls are always higher in 8-bit MCT features regardless of datasets. DE-D contains partial occlusion of traffic signs in datasets, and results show that the Byte-MCT are more robust against the noise and partial occlusion. The landmark-based AdaBoost performance for 9-bit MCT and Byte MCT result shows a different trend: both precision and recall are higher in Byte MCT feature. The margin between 9-bit MCT and Byte-MCT are even larger for noisy input especially under the low illumination inputs, i.e., KR-N.

The classifier performance between full-pixel AdaBoost and landmark-based AdaBoost, landmark-based AdaBoost always produces better performance with shorter training time. Although full-pixel based AdaBoost shows stable performance in KR-D datasets, it was largely affected by lower illumination, KR-N. Applying the landmark feature significantly enhanced performance for the partially occluded dataset, DE-D. This data depicts the situation of possible over-fitting of pixel classifiers for full-pixel AdaBoost.

Table 7 shows that Byte-MCT generate superior result as compared to 9-bit MCT. Also, Landmark-based AdaBoost performs remarkably better against partial occlusions and noise whereas full-pixel AdaBoost failed. This data leads us to believe that the proposed methods, Byte-MCT and landmark-based AdaBoost, are the best combination of methods for detection. Performances is stable regardless of driving scenes or illumination variance, even reducing the training times.

### Comparison of processing time performance

The proposed traffic sign detection and recognition method is specifically designed for parallel-window searching on a GPGPU, unlike traditional sliding-window searching. Normally, most computational processing units are CPUs, but the GPGPU allows a parallel system that is optimized for graphics and display processing, which uses thousands of processing units and pipelines. To evaluate the running time of the proposed method, we compare CPU-based operations and GPU-based operations. The input image resolution is Full-HD (1920 × 1080), which is resized to generate image pyramid of six-step down-sizing for CPU/GPGPU processing. The hardware specifications are an Intel i7-6700 3.2-GHZ CPU and an NVIDIA GeForce TITAN X GPU.

For detection stage, we use detection windows of four different sizes and experiment with both single and multiple windows on each image pyramid. Table 8 shows the CPU and GPU-based Byte-MCT transforms and detection running times. The window size is the same window size as for AdaBoost. The last row of Table 8 shows the experiment for the total processing time with all of the window sizes: four detection windows and six image pyramids. CPU-based processing takes 4,263.3ms for Byte-MCT transformation and detection, whereas GPU-based processing takes only 29.6ms, which is approximately 144 times faster than CPU-based processing. As Table 8 shows, for each task, GPGPU processing is 80 times faster than CPU processing on average.

**Table 8:** Comparison of time consumption using CPU or GPGPU.

Window Size	Image Resolution	CPU			GPGPU		
		B.MCT	Detection	Total	B.MCT	Detection	Total
		Trans.(ms)	(ms)	(ms)	Trans.(ms)	(ms)	(ms)
40 × 40	1920 × 1080	135.45	352.26	487.61	3.57	2.40	5.97
	1632 × 918	95.69	252.03	347.72	2.27	1.67	3.94
	1344 × 756	64.97	167.99	232.87	1.59	1.04	2.63
	1056 × 594	40.16	97.33	137.46	0.91	0.60	1.51
	768 × 432	20.92	49.02	69.94	0.13	0.17	0.30
	480 × 270	8.17	15.47	23.64	0.11	0.06	0.17
	**Total**	365.36	933.98	**1299.34**	8.58	5.94	**14.52**
60 × 60	1920 × 1080	135.45	333.31	452.21	3.57	2.23	5.8
	1632 × 918	95.69	248.00	343.69	2.27	1.59	3.86
	1344 × 756	64.97	152.10	217.07	1.59	1.15	2.74
	1056 × 594	40.16	86.66	126.82	0.91	0.64	1.55
	768 × 432	20.92	41.37	62.29	0.13	0.14	0.27
	480 × 270	8.17	11.02	19.19	0.11	0.09	0.2
	**Total**	365.36	872.46	**1237.82**	8.58	5.84	**14.42**
80 × 80	1920 × 1080	135.45	316.76	452.21	3.57	2.06	5.63
	1632 × 918	95.69	222.18	317.87	2.27	1.50	3.77
	1344 × 756	64.97	150.52	215.49	1.59	1.07	2.66
	1056 × 594	40.16	80.44	120.60	0.91	0.63	1.54
	768 × 432	20.92	34.31	55.23	0.13	0.17	0.30
	480 × 270	8.17	7.56	15.73	0.11	0.11	0.22
	**Total**	365.36	811.77	**1177.13**	8.58	5.54	**14.12**
100 × 100	1920 × 1080	135.45	314.14	449.59	3.57	2.01	5.58
	1632 × 918	95.69	219.28	314.97	2.27	1.45	3.72
	1344 × 756	64.97	136.07	201.04	1.59	0.94	2.53
	1056 × 594	40.16	74.25	114.41	0.91	0.71	1.62
	768 × 432	20.92	29.55	50.47	0.13	0.19	0.32
	480 × 270	8.17	4.59	12.76	0.11	0.06	0.17
	**Total**	365.36	777.88	**1143.24**	8.58	5.36	**13.94**
40 × 40	1920 × 1080	135.45	1541.65	1677.10	3.57	8.06	11.63
60 × 60	1632 × 918	95.69	1083.02	1178.71	2.27	5.76	8.03
80 × 80	1344 × 756	64.97	699.47	764.44	1.59	3.92	5.51
100 × 100	1056 × 594	40.16	367.47	407.63	0.91	2.36	3.27
	768 × 432	20.92	167.06	187.98	0.13	0.64	0.77
	480 × 270	8.17	39.66	47.83	0.11	0.32	0.43
	**Total**	365.36	3898.33	**4263.69**	8.58	21.06	**29.64**

In recognition stage, the running time is proportional to the number of verified regions from SVM, which varies across different input images. Due to this limitation, we can only compare the results based on average time consumption. The SVM and CNN run on the CPU and GPU respectively. The overall recognition takes 5ms on average, while SVM verification takes 2ms, and CNN classification takes 3ms.

## Conclusion

In this paper, we presented a GPGPU-based real-time traffic sign detection and recognition method for high resolution image inputs. This method introduces the Byte-MCT for stable candidate region detection regardless of illumination changes. For real-time high-resolution image processing, we used the target object’s landmark-point-based AdaBoost for traffic sign detection, and applied a parallel-window searching algorithm for the GPGPU. For high-performance recognition, we used a hierarchical classifier structure that combines SVM for verification and CNN for final recognition. Our proposed method makes the following three primary contributions:

1. Illumination-robust Byte-MCT and landmark-based traffic sign region detection
2. Parallel-window searching for traffic sign detection based on GPGPU
3. Hierarchical classifier structure using SVM and CNN

The presented method proposes possible solutions to two traditional challenges in traffic sign recognition.

1. Using its robustness against illumination features, the proposed method significantly improved the performance of detection and recognition under low illumination environments, i.e., nighttime.
2. The method was able to perform real-time processing using a GPGPU on full-HD resolution input.

The accuracy of proposed method is 89.5%, which is higher than the ACF result [17], 84.3%, on the VIVA test-set [18]. Furthermore, on datasets using Vienna Convention traffic rules, the proposed method performs at 97.9% accuracy on average, which is 26.8% higher than the ACF result [17], which is 71.1% on average. With a GPGPU device, DRIVER PX [3], for smart vehicles, the proposed method can be implemented in the vehicle industry in the near future.
